# Mental Model of Malaysian Pig Farmers in Implementing Disease Prevention and Control Practices

**DOI:** 10.3389/fvets.2021.695702

**Published:** 2021-11-08

**Authors:** Yong Suit-B, Latiffah Hassan, Steven Eric Krauss, Peck Toung Ooi, Siti Zubaidah Ramanoon, Abd Rahaman Yasmin, Jonathan H. Epstein

**Affiliations:** ^1^Department of Veterinary Laboratory Diagnostics, Faculty of Veterinary Medicine, Universiti Putra Malaysia, Serdang, Malaysia; ^2^Institute for Social Science Studies, Universiti Putra Malaysia, Serdang, Malaysia; ^3^Department of Veterinary Clinical Studies, Faculty of Veterinary Medicine, Universiti Putra Malaysia, Serdang, Malaysia; ^4^Department of Medicine and Surgery of Farm & Exotic Animal, Faculty of Veterinary Medicine, Universiti Putra Malaysia, Serdang, Malaysia; ^5^EcoHealth Alliance, New York, NY, United States

**Keywords:** pig farmers, Nipah, biosecurity, disease prevention and control, mental model, qualitative study, policy

## Abstract

The 1998 Nipah virus outbreak in Malaysia resulted in major financial losses to the multi-million-dollar swine industry. While productivity and biosecurity of pig farms have improved since, biosecurity in some farms remains substandard with farmers struggling to adapt to current national pig farming policies. Farm viability and animal health depends on farmers' role as decision-makers in managing disease threats and other aspects of farm management. This study aimed to describe the mental model of farmers in making decisions about disease prevention and control measures during the 1998 Nipah virus outbreak, and in 2019, 20 years after the last reported Nipah case. Using a qualitative approach, in-depth, semi-structured interviews with 12 pig farmers (mostly small-scale or medium-scale farms) were conducted in three states in Malaysia. Data were analyzed via inductive content analysis. Thirty-six unique dimensions in the mental model were identified, representing six interrelated themes corresponding to participants' decision making related to disease prevention and control: drivers of action to prevent and control disease; perception of practice options; individual determinants of familiar practices; external social factors; external economic factors; and additional external factors. Key drivers of disease control and prevention responses during the Nipah outbreak included heightened perception of risk, emotions, perceived economic loss, and subjective norms whereas key drivers in 2019 included perception of risk, perceived effectiveness, perceived benefits, and other dimensions such as perception of the future, perceived economic cost, barriers, and loss. An unfavorable future outlook, perceived economic factors, and socio-political and personal factors currently hinders farm improvement and adoption of Pig Farming Areas (PFAs) and Modern Pig Farming (MPF) systems. Private sector service providers and veterinarians are highly influential in advocating for good biosecurity, herd health, and animal health intervention practices. Insights gained can inform the development of strategic policies and interventions.

## Introduction

In Malaysia, pigs are the second largest livestock commodity with an estimated 1.7 million heads from 614 farms in 2020. The pig industry in Malaysia mostly caters to the country's ethnic Chinese population (1–3). Pig rearing in Malaysia began during the early days of Chinese settlement in the form of backyard subsistence farming. The practice flourished into a commercialized enterprise in the 1950s and then a full-fledged trade commodity by 1981 (1, 4). However, intensification of pig farms led to environmental pollution and sparked socio-religious and land development issues (5). As a solution, Pig Farming Areas (PFAs) were introduced in 1991 by the Ministry of Agriculture of Malaysia (6).

In PFAs, pig production activities were centralized in areas designated by the government to allow open and closed-house pig farms to operate. These areas have the necessary infrastructure to ensure biosecurity and waste management along with additional facilities such as laboratories, abattoirs and incinerators (6, 7). By 2007, only three out of 13 states approved PFAs due to challenges with land availability, high capital cost, disease outbreak concerns and state government by-laws. Thus, Modern Pig Farming (MPF) was introduced for states without PFAs. MPF requirements include a housing system where animals are raised in an enclosed building with zero-discharge waste management or with effluent of < Biological Oxygen Demand (BOD) 50 ppm, applies good farming practices and has a 200 m buffer zone from human habitation (8–10). Currently, a large majority of pig farms in Peninsular Malaysia are still operating as open-house systems where pigs are housed in open-sided structures (11, 12). MPF and closed-house systems will enhance productivity, efficiency, and reduce risk of disease transmission between farms or herds and between pigs and wildlife (10, 13). However, due to various challenges, farmers are reluctant to switch to MPF (14).

The 1998 outbreak of novel Nipah disease in Malaysia led to 265 cases of viral encephalitis with 109 human deaths, the culling of 1.1 million pigs and a cascading socio-economic impact (15, 16). Fruit bat is the natural reservoir of the novel Nipah virus and pigs were believed to have become infected after consuming Nipah virus contaminated fruits that fell into open pig pens (17, 18). Nipah virus causes respiratory and neurological signs in pigs with a morbidity rate of 100% and mortality rate of <5%. The virus spills from pigs to humans through direct contact, which causes similar aforementioned clinical signs. There is no vaccine or specific treatment for Nipah disease in pigs or humans (16, 18). During the 1998 outbreak, the case fatality rate in humans was high (39.6%) (15). Pig farmers in the country lived in fear as “many went to a hospital for treatment but left in a coffin” (19). Malaysia spent USD171 million on eradication and lost USD446 million from the outbreak, mostly from domestic, export and allied business (20). Pigs were culled from all 896 farms in the infected areas of Perak state, and all farms in Negeri Sembilan state and the Sepang area in Selangor state. This included all infected or uninfected farms within a 10 km radius of the outbreak. Farmers were incentivized to surrender or kill and bury their pigs voluntarily. Some farmers abandoned their farms as cases of deaths climbed and financial difficulties grew. Residents in the affected areas evacuated due to fear of the disease and impending bankruptcy. Another 50 farms in Perak, Malacca, Penang, Selangor, and Johor outside of the infected areas were identified in a subsequent national surveillance program and further culling took place in these farms (21, 22). The outbreak caused job losses to 36,000 people working in the farms and primary supporting services of the pig industry (20).

During the epidemic, emphasis was placed on training farmers across the country in early detection of disease and reporting, management of sick animals, good hygiene practices such as washing hands with soap or detergent after handling pigs, donning of protective clothing, gloves, masks, goggles, boots, long sleeves shirt, and cleaning of vehicles and equipment. All movement of pigs throughout the country as well as export of pigs to Singapore were prohibited (22, 23). Several surveillance programs found that farms that did not take in suspected infected animals tested negative for Nipah, even though they were located adjacent to an infected farm. Farms that culled grower pigs obtained for fattening rapidly from farms with suspected infection or exposure to Nipah were also free from the disease (22). The outbreak brought huge changes to the direction of the industry and greater attention to farm design, biosecurity, and environment such as monitoring the presence of fruit trees and bats around farms to prevent and minimize disease transmission (18, 23).

There were 1,885 pig farms and a 2.4 million standing pig population (SPP) prior to the Nipah outbreak. By July 1999, however, only 829 farms and 1.32 million SPP remained (16). The country's self-sufficiency level of pork fell from 137% prior to the outbreak to 79% in 2000 (1). Pig rearing was banned from outbreak areas in Negeri Sembilan state, which once had the largest pig farming community in Southeast Asia (16, 19). Pig farming was only allowed in PFAs and replacement of culled pigs was allowed only at the discretion of the state government. Farmers were encouraged to venture into other businesses (16) and agricultural sectors (19, 24, 25). Several farmers who were infected remained unemployed due to the long-term neurological effects of Nipah disease (24, 26). Ex-farm price of pigs and demand for pork struggled to return to normal in the subsequent 2–3 years after the outbreak (25, 27). Malaysia was declared Nipah free in 2001 by the Office International de Epizooties (OIE) (20). However, while serological evidence of Nipah is still found among bats in Malaysia (28, 29), Nipah disease has not re-occurred in pigs or humans since 1999.

Most pig farms are passed down from previous generations. Some aspect of the farming practices have evolved over time with training and recommendations from consultants, veterinarians, suppliers, farmer associations, and the veterinary authorities. Moreover, continuous efforts to improve biosecurity and animal health have been made through education, regular audits, certification, and advocacy for PFAs and MPF (30). However, some aspects of pig management have remained traditional; changing these type of practices is arduous and slow (31, 32). The pig industry continues to face challenges from multiple endemic swine diseases such as salmonellosis, colibacillosis, Porcine reproductive and respiratory syndrome (PRRS), sporadic cases of Classical Swine Fever and risk transboundary disease like African Swine Fever (33, 34). Farmers are at the core of farming decisions on various aspects of farm management including those that involve disease management and prevention (35). This study employed a mental model approach to describe how pig farmers' think and make decisions on the implementation of disease prevention and control practices.

### Mental Models and Farmer Decision-Making

Qualitative studies can be useful for understanding the rationale of farmers' disease prevention and control practices resolutions (36–40). Decision-making among farmers is complex and influenced by a combination of personal, business, social, emotional, motivational, and educational factors (41). Mental models are cognitive structures built on an individual or group's experiences, perceptions, and worldviews, which give precedence to reasoning, decision making, attitudes, and behavior (42). In agriculture and public health, mental models have been used to explore decision-making processes, adoption of technology, and risk communication (43–48). Individual farmers' mental models shape various aspects of farm management practice such as animal husbandry, biosecurity practices, and herd health, which subsequently influence productivity, food security, animal health, and public health (49–51). Farmers' values, beliefs and knowledge form mental models that drive action, decision-making, and use of information toward a desired definition of success (44).

Past studies on farmer's mental models have shown that mental models of farming are fortified through discovery learning, experiences and problem solving. Mental models are also continuously reinforced when they yield perceived success through resulting actions. However, a mental model can be transformed through an activating event such as a disease outbreak or through learning from another farmer's experience. Activating events challenge what farmers assume to be true, leading to self-reflection, learning, and change in perspective and farming practices (49). In the current study, a mental model approach allowed us to explore how the Nipah outbreak—which was devastating emotionally, socially and economically—impacted farmers' disease prevention and control practices and whether the impact was sustained over time.

Social factors affect decision-making. Farmers gain experience and learn to adapt farming practices or ways of managing difficulties through observation, guidance or information from family members, peer, managers, consultants, and institutions (52). Social pressure may also influence farmers to adhere to biosecurity practices (53). In healthcare, besides personal positive experience toward a health program, a family member's positive experience inspires a patient's adherence to the program illustrating a shared mental model (54). A shared mental model is molded through learning, mutual experiences, ideologies or institution and communication (55). As many Malaysian pig farmers operate multigenerational farms in close knit communities under strict laws, we expected their mental models on disease prevention and control practices to reflect intergenerational learning along with social influence from their families, communities, and institutions.

In addition to using mental models to understand decision-making, theories such as the Theory of Planned Behavior/Theory of Reasoned Action, and the Health Belief Model have been used to explore farmers' decisions and behavior pertaining to disease control (37, 38, 56, 57). The Health Belief Model (HBM) proposes that health behaviors are driven by beliefs and attitudes. Its main components include perceived susceptibility to, and severity of disease, perceived benefits of adopting a particular strategy to reduce the perceived threat of disease, and perceived barriers such as psychological, physical, or financial barriers, which maintain or suppress action. HBM suggests that self-efficacy to accomplish a behavior and cues to stimulate action are necessary. Cues to action may stem from an internal health condition or influences from health professionals, family or media that influence one's beliefs (58). This model has been used in understanding foot-and-mouth disease (FMD) control measures in cattle farmers as well as the use of personal protective equipment during pesticide handling (59, 60). The Pathway of Disease Control model, which incorporates socio-ecological elements into the Theory of Planned Behavior, was also developed to understand farmers' implementation of disease control. The model incorporates both intrinsic (attitudes, social norms, and self-efficacy) and extrinsic (community of farmer, culture and society, and access and availability of knowledge, skills, and ability) dimensions that influence the decision-making process of farmers (61). Hence, multiple theories or models can be incorporated to create the most suitable approach to understanding the drivers influencing farmers' decisions and behaviors.

### The Current Study

The conceptual framework of the current study draws on Krauss et al.'s (62) study on how Malaysian farmers form their mental models of farming, and components of the Health Belief Model (HBM) (58), to understand the precursors of pig farmers' decisions and behaviors. Due to the lack of previous research on pig farmers' disease prevention and control strategies in Malaysia, we chose to conduct the study in an exploratory manner to facilitate emergence of themes inductively. The mental model approach provided us with a broad foundation to probe the farmers' experiences during the Nipah outbreak and in the non-epidemic period. The addition of the HBM provided a framework for exploring specific health-related beliefs and intentions to provide greater focus on disease prevention and control behaviors. Understanding farmers' reasoning and motivations will help veterinarians overcome barriers to success and promote adoption of recommended disease prevention and control practices and policies. A small number of social studies on Malaysian pig farmers have focused on the socioeconomic impact and social representation of disease outbreaks and social geography (24, 63, 64). However, rationales and motivations behind pig farmers' disease prevention and control strategies have not been investigated. The current study objectives are to: (1) describe pig farmers' mental model in implementing disease prevention and control during the Nipah outbreak and 2019 (when no imminent emerging disease was present), and (2) identify other challenges related to the adherence of prescribed disease prevention and control measures.

## Methods

### Sample Characteristics and Background

Malaysia is a multiracial and multi-religious country with a total population of 32.6 million, ethnically comprised of 69% Malays, 23% Chinese, 7% Indian, and 1% others (65). Islam is the predominant religion of the country (61%), followed by Buddhism (20%), Christianity (9%), Hinduism (6%), and other religions (4%) (66). Commercial livestock industry is dominated by poultry, followed by pigs (67). Pork is consumed predominantly by non-Muslims, therefore the industry is dominated by Malaysian-Chinese.

### Site Selection

This study was conducted in Negeri Sembilan, Melaka, and Johor, which are three out of six states in Peninsular Malaysia where the novel Nipah was reported. The total number of pigs reported in these three states are 2,28,639 in Johor, 44,025 in Melaka and 241 in Negeri Sembilan (67).

### Participant Selection

The study was carried out using a qualitative descriptive approach (68). Purposive sampling was used to recruit 12 pig farmers, all of whom were farm managers or owners and principal decision makers, above 18 years old, willing to participate in the study, and able to converse in English, Bahasa Malaysia, or Chinese. In line with the qualitative research principle of sampling adequacy, the sample size was determined based on the point at which data saturation was reached (69, 70). Saturation was determined through a close reading of the data and emerging themes, whereby additional study participants did not contribute any new insights to the findings. Sampled farms were recommended by the Department of Veterinary Services, Malaysia. All participants voluntarily agreed to join the study. Interviewees were given an honorarium of MYR30 (7USD) following each interview, which is customary of such studies conducted in Malaysia (71, 72). Participants were informed that the interviewer (first author) was a Master in Veterinary Science (MVSc) degree candidate. All participants were male Malaysian-Chinese which is the dominant demography for pig farmers as the majority of farms were established by the ethnic Chinese community and are multi-generational, family-run businesses (64).

### Data Collection

In-depth, semi-structured interviews were carried out between April and July 2019 by the first author. As a veterinarian and researcher, the first author was familiar with general disease prevention and control strategies, issues surrounding execution of farm biosecurity and the tendency of local pig farmers to be sensitive in discussing disease-related issues with unfamiliar individuals. Interviews were conducted in Mandarin, the primary language of pig farmers, with occasional English and Malay. The face-to-face interviews were audio recorded and took between 15 and 60 min in the participants' homes, farms, or coffee shops. Interviews were transcribed into English by the first author who is multilingual. Vernacular expressions were maintained to preserve richness of data. An interview guide was designed by the research team, which was comprised of a sociologist, veterinary epidemiologists, a swine veterinarian and a veterinary virologist. The interview guide contained a series of open-ended questions and probes to explore motivations of disease prevention and control practices. The interviewer maintained flexibility throughout the course of the interviews to follow relevant leads in order to broaden the comprehension of issues that arise (73). The first part of the interviews was composed of general questions to build rapport. The second part probed farmers' knowledge and perceptions of zoonotic diseases and known zoonotic disease outbreaks in the past to allow farmers to reflect on Nipah, how the Nipah outbreak impacted them and changed farm management, disease prevention and control strategies, factors that influence decisions on disease prevention, treatment or control strategies, challenges and needs during a disease outbreak and after an outbreak in preventing diseases, general challenges and needs of the industry, and any related issues. Demographic questions were asked in the third part of the interview.

### Data Analysis

**Figure 1:**
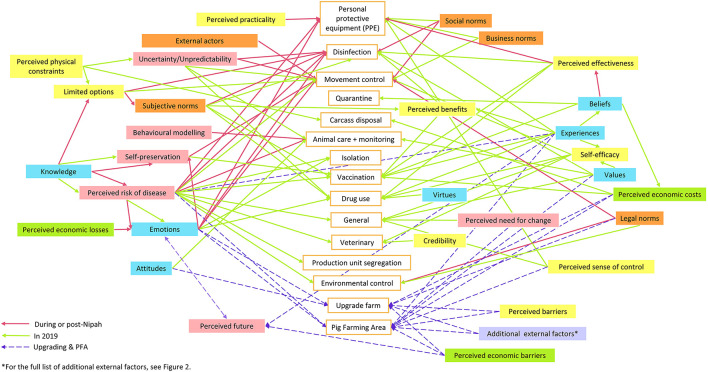
Conceptual map of 12 male Malaysian pig farmers' (10 small-medium scale farms, 2 large scale farms) mental model demonstrating relationships between dimensions and specific disease prevention and control practices. The pink arrows represent dimensions associated with practices during or post 1998 Nipah virus outbreak (practice decisions made immediately after Nipah or because of Nipah), green arrows represent dimensions associated with practices in 2019 (farmers did not expressed that practices were influenced directly by Nipah), and purple dotted arrows represent dimensions related to upgrading of farm biosecurity and/or adopt closed-house system and decision to move to Pig Farming Areas (PFAs). For example, perceived economic barriers directly influenced upgrading of farm and the decision to move to PFAs in some farmers while other farmers thought that perceived economic barriers impacted their perceived future and emotions, which subsequently influenced upgrading of farm and the decision to support PFAs.

**Figure 2:**
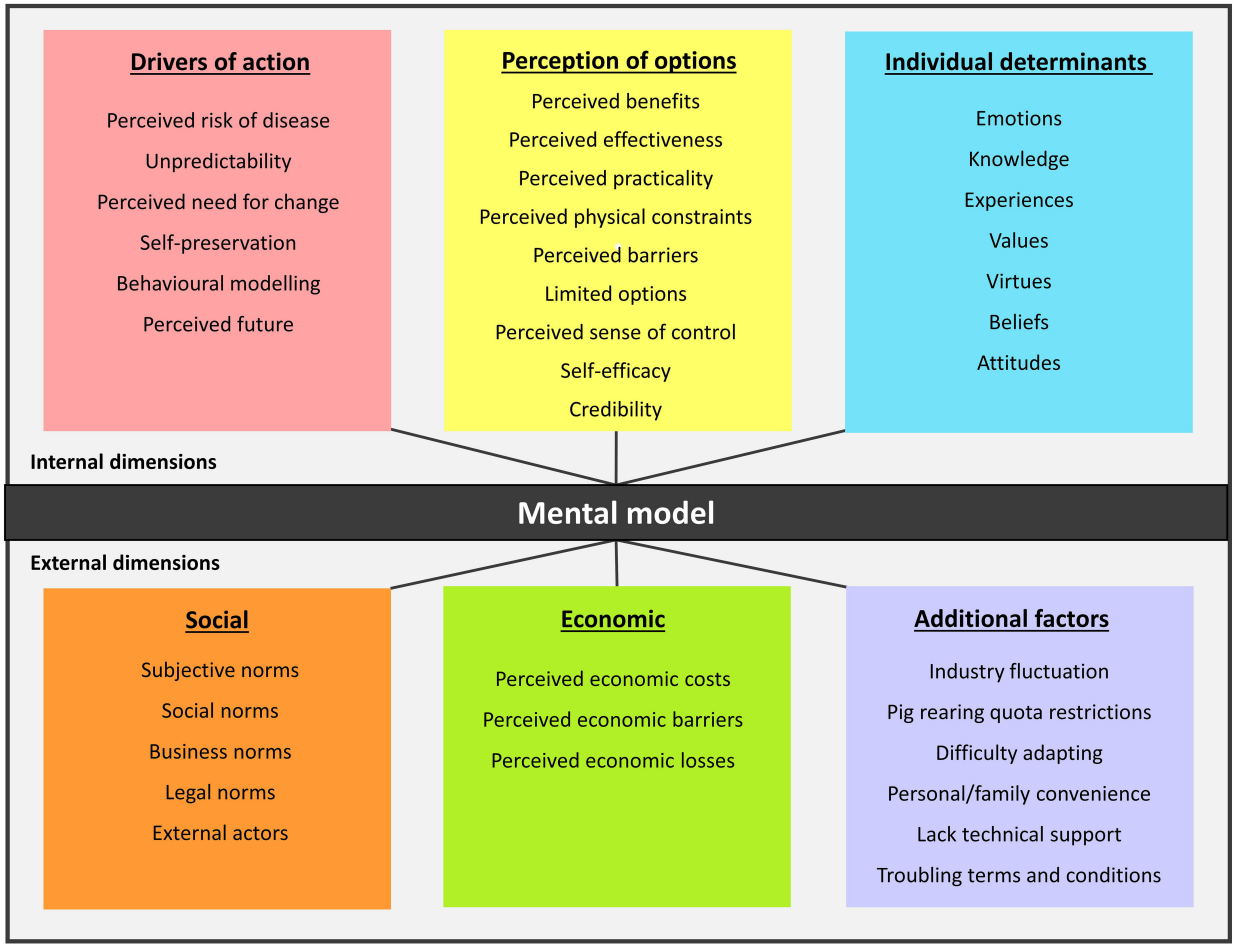
Simplified conceptual map of 12 male Malaysian pig farmers' (10 small-medium-scale farms, 2-large scale farms) mental model in implementing disease prevention and control practices during the 1998 Nipah outbreak and in 2019. The dimensions of the mental model were categorized into internal dimensions: (1) drivers of action to prevent and control disease, (2) perception of practice options which is an evaluation on options of disease prevention and control practices, (3) individual determinants which are individual factors affecting disease control and prevention choices; and external dimensions: (4) external social factors, (5) external economic factors, and (6) additional external factors.

Transcribed interviews were analyzed via inductive content analysis. This process included open coding, creating categories, and abstraction (74, 75). This form of analysis was chosen as it complements the aim of the study, which was to acquire farmers' insights on disease prevention and control practices given the limited knowledge on decision making in this community (74). All stages of the analysis were carried out by the first author under the guidance of a sociologist with extensive experience in qualitative data analysis. The sociologist was consulted on every stage of the analysis, including reviewing coded text. Through this process, coding discrepancies were resolved. Open coding was carried out by first identifying and labeling disease prevention and control practices, followed by motivations or reasons for performing or not performing those practices. Transcripts were re-read, reviewed, and coded through an iterative process. Codes and original corresponding text passages were transferred to a coding sheet and categorized into groups according to similarities. A general description of the groups was made to form dimensions of the mental model. A conceptual map was created linking each disease prevention and control practice to the codes of motivation or reasons for those practices (Figure 1) to provide a visual representation of farmers' mental processes. The process of developing the map was done intuitively, in line with Hulst et al.'s (76) approach, which incorporated a semantic web approach to the development of mental models. The current study approach differed somewhat from the work of Hulst et al. (76) by not incorporating the use of nouns to describe relationships between concepts. By demonstrating the influence between different dimensions of the mental model and their resulting practices, we also drew on the descriptive content analysis method suggested by Elo and Kyngas (74) and Vaismoradi et al. (77), who employed descriptive content analysis for health science studies. The final abstraction of the dimensions on the conceptual map was carried out to form a parsimonious conceptual map (Figure 2) with six main themes.

## Results

### Farmers' Characteristics

All farmers were Malaysian males (12/12), with ages ranging from 30 to 69 years old. Most (10/12) had a secondary education or higher (secondary education is usually completed at age 18). Nearly all (11/12) had more than 20 years of farming experience on their current farms or other farms which existed prior to the Nipah outbreak in 1998. Because of the tremendous impact of Nipah disease, all farmers in the study were highly familiar with the disease even though none reported Nipah outbreak on their farms. The severity of the outbreak left a catastrophic impact on the entire pig farming community. During and post-outbreak, all farms nationwide were on high alert and were compelled by the authorities to participate in regular disease surveillance and control activities. Furthermore, farmers who did experience Nipah first-hand were unreachable for participation in the current study as all farms that reported Nipah infection were forced to close, making tracing of those farmers impossible. Among the study sample, most (10/12) were managing open-housed farming systems, while one farmer was transitioning to MPF with closed-house system and another farmer was running a MPF or closed-house system farm at the time of the study. None of the farms were located in a PFA (Table 1).

**Table 1:** Characteristics of farms and farmers who participated in the study.

**Farm housing system^a^**	**Farm/s is located in Pig Farming Area (PFA)**	**Standing pig population (SPP)^b^, ^c^**	**Production cycle**	**Estimated years of farming experience**	**Age**	**Education**
Open	No	1,000	Closed	40	60–69	Secondary
Open	No	1,100	Closed	25	60–69	Primary
Open	No	700	Closed	20	40–49	Secondary
Open	No	500	Closed	20	60–69	Primary
Open	No	1,300	Closed	5^d^	30–39	Tertiary
Open	No	900	Closed	40	50–59	Secondary
Open	No	3,000	Closed	40	60–69	Secondary
Open	No	600	Closed	40	50–59	Tertiary
Open	No	1,000	Closed	35	40–49	Tertiary
Open	No	700	Closed	25	40–49	Secondary
Half is closed-house/transitioning to MPF^*^	No	2,000	Closed	20	40–49	Secondary
Closed-house/MPF^*^	No	13,000	Finishing	25	40–49	Tertiary

a *Farm housing system: Open-house system refers to pig farms where pigs are housed in open-sided structures. Closed-house system refers to pig farms where pigs are raised in an enclosed building. Half is closed-house refers to a pig farm that was transitioning to a fully closed-house system where half of farm has been converted to closed-house*.

b *The average SPP per farm in Malaysia is 2540 heads (5)*.

c *Classification of farm size according to SPP per farm are small (<500), medium (501–2,000) and large (>2,000) (78)*.

d *Farmer had years of non-formal farming experience from working in family farm. Farm at time of interview was his family farm that was closed for a period of time but was later revived by him 5 years before time of interview*.

* *Farmers with more than one pig farm*.

### Mental Model

Fifteen disease prevention and control practices and related issues were identified (Table 2). The reasons and motivations that made up the dimensions of the mental model were linked to specific disease prevention and control practice. Some practices were influenced by more than one dimension. At the same time, some dimensions were found to influence other dimensions, which resulted in an adopted practice. There were 36 dimensions in total (Figure 2) main themes: drivers of action to prevent and control disease, perception of practice options, individual determinants of familiar practices, external social factors, external economic factors, and additional external factors.

**Table 2:** Disease prevention and control practices identified from the interviews (10 small-medium-scale farms, 2 large-scale farms).

**No**.	**Practices**	**Category of practices**
1	Personal protective equipment (PPE)	Biosecurity practices
2	Cleaning and disinfecting	
3	Movement control	
4	Production unit segregation	
5	Environmental control	
6	Quarantine	
7	Carcass disposal	
8	Animal care and monitoring	Herd health
9	Vaccination^a^	
10	Isolation	
11	Veterinary	Animal health intervention
12	Drug use	
13	General disease control practices^b^	General
14	Upgrading farm biosecurity and/or convert to closed-house system (MPF)	Upgrading of farm
15	Decision to move to Pig Farming Area (PFA)^c^	Decision to move to PFA

a *Common vaccinations used are against diseases for Classical Swine Fever, Porcine Respiratory and Reproductive Syndrome (PRRS), Porcine Circovirus disease, and Aujeszky's disease*.

b *General disease control practices here refers to efforts to perform disease prevention and control practices that were not specified by farmers. E.g., when asked if farm's disease prevention and control practices changed over the years, a farmer replied, “Yes, when there's something to control, then there will be changes and improvements. We will only do when a disease comes, if it doesn't happen, we don't think of doing it. We will hear updates of diseases to know where it is, then we will do some”*.

c *Pig Farming Area is an area designated by state for pig farming activities under the National Agriculture Policy that compose of state-of-the-art facilities with centralized waste treatment system, breeding farm, artificial insemination centre, veterinary centre, laboratory, biosecurity, and closed-house systems. Farmers are required to purchase the lots for their farms and pay for facilities provided (7)*.

#### Drivers of Action to Prevent and Control Disease

The farmers' disease control and prevention behaviors were often influenced by their perceptions of the specific situation and environment around them, including other farmers' perceived threat of disease. Drivers of action to prevent and control disease include perceived risk of disease, unpredictability, self-preservation, perceived need for change, behavioral modeling (demonstrating good practices), and perceptions about the future. Perceived risk of disease in terms of susceptibility (outbreak is near to farm, farm is located in highly dense farming area, disease is happening or coming) or severity (disease is life-threatening, severe, zoonotic) was the most frequently referenced element driving farmers' actions around disease prevention and control during the Nipah outbreak and in 2019.

Perceived risk led to increased efforts by the majority of farmers in the study (9/12) to use personal protective equipment (PPE), undertake cleaning and disinfection, and implement movement controls and monitoring during the Nipah outbreak.

“*Nipah virus is life-threatening. We were afraid of everything. We definitely practice extra care in our personal hygiene. We are more careful. We will wear mask, use disinfectants, etc. Normally, we just wear t-shirt and pants.” (F9, 60-69 years, May 2019)*“*During Nipah, we didn't dare to buy any pigs or breeders animals. Didn't dare to buy anything. What we have was what we have. After a period of time, when things settled down, then we allowed visitors' vehicles for pig sale. We didn't dare to go to other farms either.” (F1, 60-69 years, April 2019)*

Perceived risk of disease was a main driver for practices like movement control and isolation in 2019 for some farmers (3/12) but was less emphasized as compared to during the Nipah outbreak (6/12). Practices driven by perceived risk of disease in 2019 were movement control, isolation, vaccination, production unit segregation, veterinary intervention, environmental control practices, and decision to move to PFAs.

Some farmers (3/12) expressed concerns about the close proximity of farms in a single area in 2019 and related it to their past experiences with disease outbreaks.

“*Such as Nipah in Bukit Pelandok, all the farms were concentrated at one place, then, just one infected farm, all were doomed. That's why a concentrated area is very challenging. Impossible to concentrate.” (F12, 40-49 years, May 2019)*“*With all farms in a PFA, an infected farm means all other farms were as good as gone! An infected PRRS farm was near to mine. So the wind blew to my place, my farm got infected. My farm has been infected by PRRS and Foot and Mouth Disease (FMD).” (F7, 30-39 years, May 2019)*

#### Perception of Practice Options

Perception of practice options refers to the farmers' assessment of different options for disease prevention and control practices. Dimensions in this theme include perceived sense of control, self-efficacy, benefits, effectiveness, practicality, physical constraints, barriers, limited options, and credibility. Perceived effectiveness of PPE during Nipah outbreak was frequently mentioned by farmers (6/12) while disinfection, drug and vaccination practices were mentioned in the context of 2019.

A farmer chose not to wear any PPE during the Nipah outbreak as a result of disbelief in the claimed properties of PPE and disinfection of PPE.

“*Those Tyvek suits, those masks, are all fake. In our opinion, there's no point, no matter what you do. I feel that (wearing PPE) is very excessive. If you wear, you won't be infected? I don't believe that. You wore it, come to my farm, you took it off, then you go to another farm, won't you bring the germs to other farm? I don't believe it. Surely there is – No matter how well you disinfect, germs will definitely be there.” (F7, 30-39 years, May 2019)*

Few farmers (2/12) shared that checking for antibody titres post-vaccination is an important indicator of vaccine effectiveness regardless of what pharmaceutical suppliers recommended. These farmers were willing to pay for external consultants or laboratories to run the test.

“*We don't listen to suppliers (pharmaceutical). When suppliers want to promote their products, they will first scare you. But we talk with figures. We're looking for the antibody titre which is managed by our consultants.” (F5, 40-49 years, April 2019)*“*We will only use it vaccines after a large farm uses it. This is to see the results and to consider their effectiveness. We do blood test to check for antibody titer in a private lab which we have to pay. Because suppliers have conflict of interest, hence it's impossible to rely on them to do a proper job. When there is positive titre, we will have more confidence in the product.” (F10, 50-59 years, May 2019)*

Perceived benefits (5/12) was another commonly mentioned dimension that influenced disinfection practices, herd health practices and upgrading of farm. Farmers were motivated by benefits of environmental hygiene, disinfection, appropriate air ventilation, and vaccination, believing them to be critical factors of good animal health.

“*Most importantly, a farm must be very clean. Must have sufficient water to wash. Air ventilation must be good so pigs can breathe. When it's too hot and uncomfortable for them, they fall sick. Just like humans, if your environment is good, you don't have to see doctor. That's why we rarely need to use drugs in the farm. We disinfect every transport that comes in not because of any particular disease (Nipah). We do it all the time.” (F12, 40-49 years, May 2019)*“*Vaccination program is compulsory, like for Aujezsky. You can't do without it. It's good to help control diseases.” (F10, 50-59 years, May 2019)*

One farmer expressed his desire to upgrade to a closed house system for the benefits of greater environmental control, labor saving technology, more efficient production, and less environmental pollution.

“*I really want to upgrade to a closed house system. It is good because you can manage your environment properly with the cooling system, thus better animal performance. I've seen some farms that uses a feed pump to send feed all at once to different sizes of pigs which saves labour. When you invest in such facilities, there will be less problems. Environmental pollution is better controlled and business return is faster because production will be better than open housed systems.” (F7, 30-39 years, May 2019)*

#### Individual Determinants of Familiar Practices

Individual determinants of familiar practices are personal, intrinsic factors affecting disease control and prevention choices. They include personal values, virtues, beliefs, attitudes, emotions, knowledge, and experiences that can also result in the impulse to adopt certain disease control and prevention practices. ‘Emotions' was the most recurring dimension in this theme (12/12) and affected various practices during Nipah and in 2019. During the Nipah outbreak, emotions strongly influenced PPE practices, and disinfection and movement control, while in 2019, drug use, isolation practices, upgrading of farm, and decision to move to PFAs were the most salient factors. Farmers' sentiments of perceived risk of disease, economic loss, and self-preservation were mostly coupled with emotions of fear and worry during the Nipah outbreak (8/12) and in 2019 (2/12).

“*Definitely (I) felt afraid and worried during Nipah. Not only that, your life is threatened and you can go bankrupt. And there were no drugs. And if something happens, government will cull all the pigs. Everything will be gone.” (F10, 50-59 years, May 2019)*“*When an animal become sick, we feel scared, because they are one of our property. When they die, our money will disappear too.” (F10, 50-59 years, May 2019)*

Disinfectants effectively inactivate Nipah virus (79). However, one farmer who did not believe in the importance of disinfectants continued to use them on his farm during the Nipah outbreak for his own peace of mind. The farmer thus took appropriate action despite his beliefs.

“*Disinfect. Many of us do disinfection. Actually, if it's a virus, disinfection is not effective. It only gives us peace of mind. No matter what, a vaccine is needed.” (F3, 40-49 years, April 2019)*

Emotions in the form of fear and anxiety related to farm closure or relocation to PFAs hindered many farmers from upgrading their farm to closed-house systems (8/12). These emotions were greatly fuelled by a dimension from another theme—perceived future. Fear and pessimism about the future was due to several factors including: uncertainty as to whether annual licenses would be renewed; perceived preference of state veterinarian authorities for larger farms with better waste management; perceived lack of political will by state authorities for national pig farming policies to designate a PFA or upgrading of current farms to closed-house system; continuous urban development near farms that would lead to public complaints in the future; unpleasant experiences and loss of previous PFA investments (e.g., purchased land in PFA but the project was terminated by local authorities due to vicinity to the airport); and skepticism about support for the pig farming industry. As a result, a few of the farmers (2/12) expressed emotions of helplessness and frustration due to their inability to upgrade their farms as state and local authorities halted their plans, further risking public complaints and high fines in the future.

“*The future of the pig industry is not optimistic. If possible, I would not stay in this industry. There's a pig farming area, but no one wants to go there because it includes costs. Sooner or later, the authorities will destroy that area. Pig farms in this state are also decreasing.” (F11, 40-49 years, April 2019)*

*“First this minister say he wants this, then when there's a change in minister, he says no to closed-house, we want to do another thing. So how? Like here now, he said you can farm at your present place, but they don't encourage modern closed-house pig farms although federal government said okay. We had a dialogue with the state government. They cannot guarantee it! They cannot give us the time. Say, if they gave us 30, 40 or 50 years, we can run this pig farm. Because if we invest, we're not just investing tens of thousands, its millions!” (F10, 50-59 years, May 2019)*

#### External Social Factors

External social factors comprised a variety of socially normative practices and policies that influence farmers' decision-making. These included: subjective norms—perceived social pressure by an important person to perform or not perform a particular behavior (80); legal norms—the civil or municipal law that regulates conduct (81); social norms—a conditional preference for conformity that is dependent on relevant expectations (82); business norms—organizational standards or culture that employees are expected to follow; and key external actors in the work environment. Subjective norms, such as those practiced by veterinary pharmaceutical suppliers, farm consultants, veterinary authority, superiors, or family members were influential to many farmers (7/12). Among the different types of subjective norms, suppliers' advice was most influential on farmers' (7/12) disinfection, drug use, and vaccination practices. Disinfection advice from suppliers during Nipah was particularly helpful to convince farmers (5/12) to disinfect. When asked how he knew disinfection was an effective practice during disease outbreak, the farmer replied, “I heard from the feed seller that this is a good practice. It's more hygienic this way” (F4, 60–69 years, April 2019). Suppliers also influenced a skeptical farmer about the efficacy of disinfection during the Nipah outbreak.

“*They (pharmaceutical suppliers) can't do anything even if they come because they don't have the drugs (to treat Nipah). So they will ask to disinfect more.” (F3, 40-49 years, April 2019)*

In 2019, one farmer relied on farm consultants to guide vaccination practices, while a farm manager mentioned that he minimized the use of drugs because of his boss' insistence on practicing good virtues to raise healthy animals for human consumption.

“*He says that as a businessmen, we need to be kind, honest and sincere. This means having good virtues. He reminded us that we must rear healthy pigs for people to consume. Yes, that's what Chinese call as honesty. Do things with good virtues, not evil.” (F5, 40-49 years, April 2019)*

#### External Economic Factors

The economic component was one of the most prevalent factors among farmers (10/12) in facilitating disease prevention and control practices during Nipah in 1998 and during periods with no imminent emerging disease threats such as in 2019. Perceived economic losses due to disease outbreak, culling of pigs, bankruptcy and loss of livelihood prompted some farmers (3/12) to disinfect frequently and control the movement of people, vehicles, and pigs in and out of their farms, especially during the Nipah outbreak. Although the Nipah disease was zoonotic, one farmer (1/12) chose not to practice PPE in 1998 due to perceived impracticability and felt that “he had nothing to lose.” When asked his reasons for not using PPE, the farmer replied, “Because I don't have money (laughs). No money, not afraid to die. Die faster, the better, right?” (F3). One farmer was concerned with the reduced profitability and control over certain farm management aspects in PFAs and therefore chose not to move to a PFA in 2019.

“*We can't move this farm there, we will need to have a whole new batch of pigs. Feed has to go through to them (PFA management). Water, you have to pay. Everything, everything is also controlled by them. That's why you're doomed, unless you can control the buying and selling price of pork out there.” (F12, 40-49 years, May 2019)*

Perceived economic costs was the second component that guided farmers' (5/12) decisions on vaccinations (purchasing cost, selective vaccinations) and carcass management (preference to burn the carcasses manually rather than buy an incinerator), resulting in farmers' hesitation to upgrade farm biosecurity, move to a closed-house system, or move to a PFA in 2019. Farmers (7/12) expressed the desire to upgrade but could not due to inadequate funds and financial support such as low interest rate loans and subsidies, and lack of support to increase number of pig rearing quotas that will allow return after high-cost investments. Fear of not being compensated for farm closure or relocation was also an issue that increased farmers' reluctance.

“*For me, okay to follow the government policy of doing a closed-house system, that's our national agriculture policy, our mission and motto. But in the end, its only talk as there's no budget for the pig industry. The budget goes to chicken, cattle, goats. None for pigs. One more thing, we called (Bank A). (Bank A) doesn't want to lend to non-halal business. That's a problem. We would like a lower interest rate (for loans). But where to get the money? If we want to do large-scale, to upgrade, we need government subsidies.” (F10, 50-59 years, May 2019)*

“*Closed-house is good. The problem is that the cost is too big. If the authorities let us rear a few thousand pigs like large farms, then you can have this kind of facility. We rear only few hundred, and you ask us to do a proper closed-house system? We need more than a million. If we only sell a few pigs a month, how long will it take for us to have returns? We can't get returns. Building a closed house system is not profitable! We want to rear more (pigs), but the authorities won't permit it, then how do we go about it?” (F12, 40-49 years, May 2019)*

#### Additional External Factors

Additional external factors guiding farmers' decision-making in 2019 included: industry fluctuation and pig rearing quota restrictions, which affected profitability and financial capability to improve farms; perceived challenges of adapting to new farms in PFAs; traveling distance and children's schooling as a deterrent to move to PFA; perceived lack of technical support; and troubling terms and conditions for building closed-house system in 2019.

A farmer shared that high farming costs coupled with low market price of pork resulted in minimal profits, which is inadequate for upgrading of farms.

“*If you need a lot of funds (to upgrade farm), then we don't have enough. Because the pork prices are bad recently, feed is also expensive. After deducting living expenses, staff wages and maintenance, there's not much profit left.” (F9, 60-69 years, May 2019)*

The inconvenience caused to the farmers and their families due to traveling long distances between home and the location of the PFA, along with the difficulties that adapting to the PFA, acted as a deterrent to moving to a PFA according to one farmer.

“*What about our children's school? Going there from the farm is also (very far). Now, my family is still with me. But here to there (the farm) is about 3 hours, then back will be 6 hours. Every day back and forth, I'll be exhausted…. Not only that, new place, new things, we are also not used to it. So even there's a pig farming area there, no one wants to move there.” (F11, 40-49 years, May 2019)*

## Discussion

Through exploring the rationale of farmers' decision-making and the adoption of disease prevention and control practices, six interrelated themes comprised the farmers' mental model: drivers of action to prevent and control disease; perception of practice options; individual determinants of familiar practices; external social factors; external economic factors; and additional external factors were determined. Our findings indicated that decision-making and subsequent behavioral responses in perilous situations like Nipah were unlike those for day-to-day practices. Farmers were more vigilant in practicing PPE, cleaning and disinfecting, practicing stricter movement control, and monitoring of animals during the Nipah outbreak as compared to 2019 when no imminent epidemic threat existed. Elevated responses for these practices were mostly influenced by heightened sensitivity of disease risk and fear of catastrophic outcomes to their businesses and livelihoods. These factors activated self-preservation and amplified biosecurity practices. Additional precautions were more likely to be employed by farmers when they perceived disease to be severe or when they perceived the threat of outbreak imminent, especially if a neighboring farm was infected. Past studies have reported that farmers' risk assessment is often based on a combination of knowledge and experience, which includes an understanding of their own farm, neighboring farms and possible routes of transmission (37, 83). In Mankad's (53) review on the psychological influences of biosecurity control and farmers' decision making, the author suggested that fear of disease threat can drive behavioral modification, or feelings of denial and avoidance. Our study demonstrated that in addition to perceived risk, stress, and fear were motivating factors for elevated biosecurity measures during the Nipah outbreak. Alarcon et al. (38) reported that in situations of despair, farmers may act against veterinary advice and conform to other farmers' advice or accept veterinary advice albeit minimal data or uncertain level of effectiveness. In our study, despite one farmer's erroneous belief in the ineffectiveness of disinfection, the farmer practiced disinfection for his own peace-of-mind during the Nipah outbreak. This farmer's action, along with others in the study, was also influenced by suppliers' advice to disinfect during the Nipah outbreak.

Past experience of an outbreak can cause prolonged social and psychological effects that may impact subsequent behavior (84, 85). In the UK, for instance, a study found that farmers were still cautious of FMD even after 4 years of the FMD outbreak when purchasing stock from other farms (86). Although farmers in our study expressed fear of reliving a similar outbreak like Nipah after 20 years, this fear did not help to preserve certain biosecurity practices, such as wearing PPE or designated farm clothes and frequent cleaning and disinfection due to the practical concerns of running their farms in 2019. This may be also be influenced by the difference in perception of endemic diseases vs. new epidemic diseases like Nipah. In support of our findings, Valeeva et al. (57) reported that farmers perceived epidemic diseases to be more deleterious to the economic performance of the farm as compared to endemic diseases. The authors concluded that the lower perceived susceptibility of endemic diseases directly influenced the lack of implementation of risk management strategies (57). In our study, farmers were more apathetic about PPE and hygiene practices in non-urgent periods where susceptibility to serious diseases such as Nipah were absent.

Farmers understood and valued evidence-based strategies to protect their herds from disease. Perceived effectiveness and perceived benefits were predictors of whether farmers adopted a certain practice. It is important to note that farmers were skeptical of pharmaceutical suppliers' sales pitches about vaccines despite expressing trust in other disease prevention and control advice. This finding corresponds with other reports where farmers were unlikely to proceed with a practice if evidence of effectiveness was absent. In studies by Alarcon et al. (38) and Valeeva et al. (57), the authors reported that farmers adopt strategies that they believe to be effective, which is influenced by other farmers' positive experience and veterinary advice. The lack of perceived effectiveness in our study could have stemmed from insufficient knowledge, belief or experience, as shown in Figure 1. Therefore, extension services can provide clearer and more detailed information to promote disease prevention and control practices. Nonetheless, according to Ritter et al. (87), feasibility and practicality are also important considerations for farmers.

The findings suggest that a pessimistic view of the future demotivated farmers to upgrade their farms or move to PFAs. Most farmers desired to upgrade their farms, but were constrained by a lack of financial resources, institutional and logistical support, and lack of political will. Low farm-rearing quotas were viewed as insufficient to sustain farmers' businesses after high investment costs and risk of closure without compensation during the building process or before a return of investment is achieved. Liu (88) reported a case of a farmer who invested several hundred thousand Malaysian Ringgit on waste-treatment facilities but was later instructed that he could only rear a low number of pigs, making the business non-viable. Thus, farmers' worries are justified especially when farming is a source of livelihood. Moreover, lack of confidence in the authorities due to perceived pressures of farm closure or mandatory relocation to PFAs, and the farmers' opinion that the authorities and local community do not support pig farming further discouraged farmers to make improvements or investments. In line with previous studies, feelings of inadequate institutional support can diminish farmers' perceived responsibility and subsequent attempts to employ disease prevention and control efforts (87). Although the general rationale for the Malaysian government pig rearing quota is well-supported for reasons of food safety, animal welfare, environmental preservation, and pollution control (89–91), this rationale seems less tangible to farmers operating with marginal profits. The situation described in our study mirrored the sentiment by the local livestock federation that pig farming is increasingly difficult to operate and more proactive government support is needed to ensure farm sustainability (92).

Farmers' antagonistic view of PFAs in our study was shaped by their observations, memories, and perceptions that Nipah was transmitted between farms located close together in the PFA at Bukit Pelandok. All pigs in the infected and surrounding areas were destroyed as an eradication measure during the Nipah outbreak, causing major losses to farmers (20, 22). Even though the employed measures are a standard recommendation by international bodies for controlling disease spread (79), it is likely that farmers with significant emotional and economical attachment to their farms find these measures difficult to accept. As reported in previous studies, in addition to concerns about the economic impact of the Nipah virus, the outbreak also had a significant emotional impact, subsequently affecting the pig farmers' practices (38, 84). Economic viability is an important factor in farming (38, 93) and this dimension was prevalent in our study for many practices.

Social factors are widely recognized as influencing livestock farmers' decisions (94). Our study found that subjective norms or perceived social pressures to perform a behavior were the most common elements that arose in deciding disease prevention and control practices. Suppliers' recommendations were taken seriously and more often yielded positive behavioral outcomes. These suppliers were commonly veterinarians from private pharmaceutical or feed companies that had established relationships with the farmers. Other authors (36, 95) have also reported that farmers are more receptive to advice given by suppliers than from local authorities in the current study, only one farmer mentioned the role of the veterinary authorities as a factor in practicing disinfection. The finding advocates for more public-private agency collaboration in advising farmers and disseminating accurate and updated information about farm management, disease prevention and control, and pig farming policies. Suppliers should also be included in policy discussions about direction of the industry to leverage their strong relationship with the farmers. In addition, other studies have also found that instilling good values and interpersonal skills in veterinary training can significantly improve veterinarians' ability to indirectly change the mindset of farmers and enhance behavioral change around disease prevention and control (96, 97).

Given the inherent limitations of the study design, findings must be interpreted with caution. None of the farmers interviewed reported Nipah infected pigs during the outbreak in 1998 and a few of the farmers were young. However, the risk and impact of the Nipah outbreak was significant and pervasive to the country's pig farming community. Even those farmers with non-infected farms were deeply affected. Younger members of the farming community may or may not have contributed to decision-making during the outbreak in 1998, however, most were able to vividly recall many details about the outbreak and how those experiences shaped their current practices. Although positive and negative influences of each dimension were determined during coding to create the conceptual map and discuss in the results, these was not labeled on the map to avoid overcrowding. Interviews were conducted with a small sample of 12 pig farmers, most of whom run small to medium-scale farms. Accordingly, it is not possible to generalize the findings to all pig farmers in Malaysia. Yet, an explorative study like this provides critical insights to pig farmers' judgment and motivations, decision-making processes and external constraints and can act as a guide for larger studies in the future.

## Conclusion

The findings suggest that imminent disease outbreak threats increase pig farmers' vigilance toward biosafety and biosecurity practices including PPE, cleaning and disinfecting, and movement control. This heightened vigilance is likely driven by disease risk and fear of catastrophic outcomes to the farmers' businesses and livelihoods. Our findings also indicate that in order to improve the implementation of local disease control policies, the farmers' worldviews, practical challenges and motivation for decision-making need to be better understood to ensure support and timely execution of the policies. We advocate for better engagement between the veterinary authorities and stakeholders in the pig farming support industries. These stakeholders have close relationships with pig farmers and may be able to identify better avenues for delivery of knowledge, taking into account farming community sentiments and priorities. From this engagement, more pragmatic and realistic animal health programs and policies can be strategized.

## Data Availability Statement

The datasets presented in this article are not readily available because of ethical restrictions to protect confidentiality of respondents. Requests to access the datasets should be directed to Steven Eric Krauss, lateef@upm.edu.my.

## Ethics Statement

The study was approved by the Ethics Committee for Research Involving Human Subjects, Universiti Putra Malaysia. The participants provided their written informed consent to participate in this study.

## Author Contributions

YS-B, LH, and SK: conceptualization, methodology, and project administration. YS-B: data curation, formal analysis, investigation, resources, visualization, and writing—original draft. LH, SK, and SR: supervision. YS-B and SK: validation. YS-B, LH, SK, PO, SR, AY, and JE: writing—review and editing. All authors contributed to the article and approved the submitted version.

## Conflict of Interest

The authors declare that the research was conducted in the absence of any commercial or financial relationships that could be construed as a potential conflict of interest.

## Publisher's Note

All claims expressed in this article are solely those of the authors and do not necessarily represent those of their affiliated organizations, or those of the publisher, the editors and the reviewers. Any product that may be evaluated in this article, or claim that may be made by its manufacturer, is not guaranteed or endorsed by the publisher.
