# Supplementary material for: Mental Model of Malaysian Pig Farmers in Implementing Disease Prevention and Control Practices
Source: Front Vet Sci. 2021 Nov 8;8:695702. doi: 10.3389/fvets.2021.695702 (PMC8606826; doi:10.3389/fvets.2021.695702)
Supplement: Supplementary file 1 [file Data_Sheet_1.docx]

**Interview Guide for In-Depth Interviews with Pig Farmers**

Ice-breaking questions

1. How many years have you reared pigs?
   你养了多少年猪？
2. How many livestock are there in your current farm?
   你现在的农场里有多少只猪？
3. What is the purpose of the livestock?
   这些猪会用于什么?

Questions

1. Can you tell me what do you know about zoonotic diseases @ diseases that can be transmitted between animals and humans?
   您能告诉我你对人畜共患疾病有什么了解吗？@可以在动物和人类之间传播的疾病?
2. Have you had any seminar or courses on zoonoses?
   Besides seminar/courses, where else do you get information from?
   你有没有参加过人畜共患疾病的讲习会或课程吗？
   除了讲习会或课程, 您从哪里得到信息?
3. What are the zoonotic disease outbreaks that you are familiar with or have experienced?
   If no, have your friends experienced any diseases transmitted from animals?
   (reflect on common zoonotic disease outbreaks in the past)
   您能分享您熟悉或经历过的人畜共患疾病爆发吗？
   如果你没有经历过, 您的朋友有吗？
4. How did the outbreak affected you?
   (probe: emotionally, financially, husbandry decisions, farm management, personal hygiene)
   疾病爆发是如何影响你呢?
   (probe: 情感上，经济上，畜牧业，农场管理，个人卫生)
5. Can you tell me about what do you do to prevent and control disease in your farm?
   您能告诉我您在农场中如何预防和控制疾病吗？
   Has it changed over the years? (If yes – what are some of the changes, if not, probe why not)
   这些年来, 这些预防和控制计划有变化吗? (如果有,-是什么变化? 如果没有, 为什么没有改变呢?)
6. What are the factors that you consider in deciding a disease prevention and control program or action?
   在决定疾病预防和控制计划或行动时，您会考虑哪些因素？
7. What are the challenges or limitations and needs during and after a disease outbreak?
   在疾病爆发期间和之后, 你有哪些挑战或限制和需求?
8. Is there anything else that you would like to add to the topic that we’ve discussed today?

您还有什么要分享关于今天讨论的话题吗？

# Demographic

1. What is your age?
   请问您的年龄范围是多少?
   1. 20-29
   2. 30-39
   3. 40-49
   4. 50-59
   5. 60-69
   6. 70-79
2. Sex
   性别
   1. Male 男
   2. Female 女
3. What is your education level? (optional)
   请问您的教育水平是多少？
   1. Illiterate 文盲
   2. Literate 识字
   3. Primary 小学
   4. Secondary 中学
   5. University 大学
